# How are ECG parameters related to cardiac magnetic resonance images? Electrocardiographic predictors of left ventricular hypertrophy and myocardial fibrosis in hypertrophic cardiomyopathy

**DOI:** 10.1111/anec.12763

**Published:** 2020-04-23

**Authors:** Zsofia Dohy, Andras Vereckei, Viktor Horvath, Csilla Czimbalmos, Liliana Szabo, Attila Toth, Ferenc I. Suhai, Ibolya Csecs, David Becker, Bela Merkely, Hajnalka Vago

**Affiliations:** ^1^ Heart and Vascular Center Semmelweis University Budapest Hungary; ^2^ 3rd Department of Internal Medicine Semmelweis University Budapest Hungary

**Keywords:** cardiac magnetic resonance, electrocardiography, fragmented QRS, hypertrophic cardiomyopathy, myocardial fibrosis, strain pattern

## Abstract

**Background:**

Structural myocardial changes in hypertrophic cardiomyopathy (HCM) are associated with different abnormalities on electrocardiographs (ECGs). The diagnostic value of the ECG voltage criteria used to screen for left ventricular hypertrophy (LVH) may depend on the presence and degree of myocardial fibrosis. Fibrosis can cause other changes in ECG parameters, such as pathological Q waves, fragmented QRS (fQRS), or repolarization abnormalities.

**Methods:**

We investigated 146 patients with HCM and 35 healthy individuals who underwent cardiac magnetic resonance imaging (CMR; with late gadolinium enhancement [LGE] in HCM patients) and standard 12‐lead ECGs. On the ECG, depolarization and repolarization abnormalities, the Sokolow–Lyon index, the Cornell index, and the Romhilt–Estes score were evaluated. The left ventricular ejection fraction, volumes, and myocardial mass (LVM) were quantified. Myocardial fibrosis was quantified on LGE images.

**Results:**

The sensitivity of the Romhilt–Estes score was the highest (75%), and this hypertrophy criterion had the strongest correlation with the LVM index (*p* < .0001; *r* = .41). The amount of fibrosis was negatively correlated with the Cornell index (*p* = .015; *r* = −.201) and the Sokolow–Lyon index (*p* = .005; *r* = −.23), and the Romhilt–Estes score was independent of fibrosis (*p* = .757; *r* = 0.026). fQRS and strain pattern predicted more fibrosis, while the Cornell index was a negative predictor of myocardial fibrosis (*p* < .0001). Among others, the strain pattern was an independent predictor of the LVM (*p* < .0001).

**Conclusion:**

The Romhilt–Estes score is the most sensitive ECG criterion for detecting LVH in HCM patients, as myocardial fibrosis does not affect this criterion. The presence of fQRS and strain pattern predicts myocardial fibrosis.

## 1 INTRODUCTION

Hypertrophic cardiomyopathy (HCM) is a primary myocardial disease with diverse morphological and clinical presentation. As HCM is considered as one of the most common causes of sudden cardiac death in young people (Maron, Haas, Ahluwalia, Murphy, & Garberich, 2016), its early diagnosis and exact risk stratification are critically important. Although the European and American guidelines on HCM highlight the importance of electrocardiographs (ECGs) in the screening and early diagnosis of patients, little is known about the specific ECG criteria of the disease and the relations of ECG criteria to histological characteristics (Elliott et al., 2014; Gersh et al., 2011). The typical pathological features of HCM include myocyte disarray, small‐vessel disease, and myocardial fibrosis, which usually have a patchy mid‐myocardial distribution in the hypertrophic segments (Varnava, Elliott, Sharma, McKenna, & Davies, 2000). Cardiac magnetic resonance (CMR) is the gold standard method for detecting and quantifying myocardial fibrosis (Moon et al., 2004). Moreover, CMR provides accurate information about the wall thickness, ventricular volumes, mass, and ejection fraction.

Structural myocardial changes in HCM are associated with electrical abnormalities. On a standard 12‐lead ECG, non‐specific patterns can be detected, such as signs of hypertrophy, pathological Q waves, and ST‐ and T‐wave abnormalities (Elliott et al., 2014; Hancock et al., 2009). The diagnostic accuracy of the different conventional ECG hypertrophy criteria (the Cornell index, the Sokolow–Lyon index, and the Romhilt–Estes score) seems to be variable in patients with HCM according to different studies (Charron et al., 2003; Delcre et al., 2013; Erice et al., 2009). The ECG voltage can be affected by the degree of myocardial hypertrophy and the extent of fibrosis. Fibrotic tissue is electrically inert and may reduce the ECG voltage, which could explain the varied and relatively low sensitivity of ECG criteria for left ventricular hypertrophy (LVH).

Pathological Q waves are considered a marker of myocardial scarring, although in HCM, pathological Q waves seem to be generated by asymmetric hypertrophy rather than by myocardial fibrosis (Fronza et al., 2016). Even in patients with prior myocardial infarctions, the overall sensitivity of the presence of Q waves is limited; in the case of a lateral infarction, this sensitivity is approximately 25% (Das, Khan, Jacob, Kumar, & Mahenthiran, 2006). Previous studies suggest that fragmented QRS (fQRS) complexes are more sensitive than pathological Q waves for detecting regional myocardial scarring in patients with coronary artery disease (Das et al., 2006; Pietrasik & Zareba, 2012). The diagnostic value of fQRS for the detection of myocardial fibrosis in HCM has not yet been elucidated; however, some data suggest that the presence of fQRS shows better diagnostic accuracy than pathological Q waves for detecting myocardial fibrosis in HCM (Konno et al., 2015). Due to myocardial fibrosis, not only depolarization but also repolarization can be affected (Sakamoto et al., 2015), which can be detected on ECGs as ST‐T abnormalities or strain pattern (Ogah et al., 2008).

The aim of our study was to investigate the diagnostic accuracy of ECG hypertrophy criteria using CMR to diagnose LVH and the impact of myocardial fibrosis on these criteria and to define ECG predictors of LVH and myocardial fibrosis in patients with HCM.

## 2 METHODS

### 2.1 Study population

We enrolled 146 HCM patients who underwent CMR examinations and a standard 12‐lead ECG at the Heart and Vascular Center of Semmelweis University Budapest. We did not enroll patients with confounding comorbidities, such as untreated hypertension, significant aortic stenosis, previous myocardial infarction, and patients who did not receive contrast agents because of severely reduced kidney function (glomerular filtration rate <30 ml/min/1.73 m^2^), did not provide consent, had undergone prior surgical myectomy or percutaneous transluminal septal myocardial ablation, or with persistent ventricular stimulation by an implanted pacemaker.

A control group of 35 healthy individuals without any known cardiovascular diseases was selected; these individuals underwent non‐contrast CMR examinations and a standard 12‐lead ECG at the Heart and Vascular Center of Semmelweis University Budapest.

Ethical approval was obtained from the Hungarian National Institute of Pharmacy and Nutrition (OGYEI/29174‐4/2019), and this study was performed in accordance with the ethical standards in the 1964 Declaration of Helsinki and its later amendments.

### 2.2 Cardiac magnetic resonance

Cardiac magnetic resonance examinations were conducted with a 1.5 T magnetic resonance (MR) scanner (Achieva, Philips Medical Systems) using a 5‐channel cardiac coil*.* Retrospectively gated balanced steady‐state free precession cine images were acquired in 2‐chamber, 4‐chamber, and left ventricle (LV) outflow tract views. Additionally, short‐axis images with full coverage of the LV were obtained. LGE imaging was performed in HCM patients after they had given their informed consent. As the control patients were free of complaints and clinical cardiovascular disease, they did not receive contrast agents because of ethical considerations. During an expiratory breath‐hold, a bolus of gadobutrol (0.15 mmol/kg) was injected at a rate of 2–3 ml/s through an antecubital intravenous line. LGE images were acquired using a segmented inversion recovery sequence with additional phase‐sensitive reconstructions in the same views used for cine images 10–20 min after contrast administration.

**FIGURE 1:**
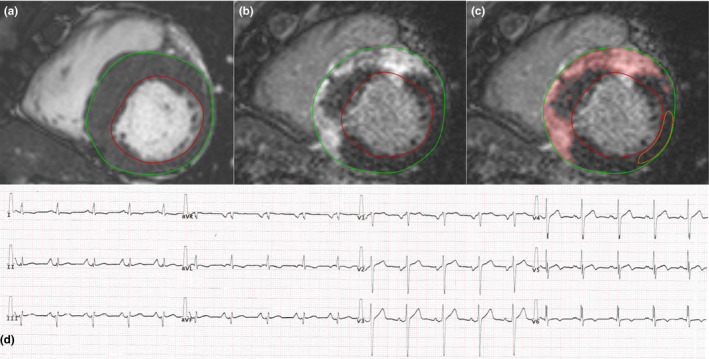
The bSSFP cine (a) and delayed contrast enhancement (b and c) short‐axis images in the end‐diastolic phase and the ECG record (d) of a patient with septal HCM. (a) Evaluation of LVM with endo‐ and epicardial contours (LVM 200 g, LVMi 90 g/m^2^, maximum wall thickness 26 mm). (b and c) Quantification of myocardial fibrosis on LGE images. The amount of myocardial fibrosis was 96 g, which was 48% of the LVM. (d) Standard 12‐lead ECG. fQRS was observed in leads I, II, aVR, aVL, aVF, V4‐V6. Strain pattern was observed in leads I and aVL. Sokolow–Lyon index = 2.4 mV, Cornell index = 2.4 mV, and Romhilt–Estes score = 7, so only the Romhilt–Estes score was diagnostic for LVH. bSSFP, balanced steady‐state free precession; ECG, electrocardiograph; fQRS, fragmented QRS; HCM, hypertrophic cardiomyopathy; LGE, late gadolinium enhancement; LVH, left ventricular hypertrophy; LVM, left ventricular ejection fraction, volumes, and myocardial mass; LVMi, left ventricular mass index

Cardiac magnetic resonance data were analyzed using Medis QMass 7.6 software (Medis Medical Imaging Software). The left ventricular ejection fraction, volumes, and myocardial mass (LVM) were quantified. Left ventricular volumes and the LVM were standardized to the body surface area (BSA). Maximal end‐diastolic wall thickness measurements were taken in a short‐axis slice perpendicular to the myocardial center line, excluding trabeculations. On LGE images, myocardial fibrosis was quantified at a grayscale threshold of 5 standard deviations (*SD*s) above the mean signal intensity for normal myocardium (Maron, 2013) (Figure 1). Semiautomated quantification of the myocardial fibrosis was visually controlled, and obvious artifacts were corrected.

To investigate the reproducibility of the quantification methods used, two independent observers (an experienced reader who had evaluated more than 500 individual original CMR cases and an unexperienced reader who had evaluated approximately 50 CMR cases) evaluated CMR images in a subgroup of 30 HCM patients and quantified the myocardial fibrosis.

### 2.3 Electrocardiography

A standard 12‐lead ECG (25 mm/s and 10 mm/mV) was obtained while all patients were in a supine position during quiet respiration. We assessed LVH with the Cornell index (positive score ≥2.8 mV for men and ≥2 mV for women), the Sokolow–Lyon index (positive score ≥3.5 mV), and the Romhilt–Estes score (4 for probable LVH and ≥5 for definitive LVH) (Casale et al., 1985; Romhilt & Estes, 1968; Sokolow & Lyon, 1949).

Pathological Q waves were diagnosed if the Q wave was ≥0.04 s in duration or deeper than 1/4 of the following R wave in at least two contiguous leads except the aVR lead.

Fragmented QRS was defined as in previous studies (Das et al., 2006; Konno et al., 2015; Pietrasik & Zareba, 2012): the presence of an additional R wave (R′), notching of the R wave or notching in the nadir of the S wave in two contiguous leads in patients with a QRS duration <120 ms. In patients with bundle branch block (a QRS duration ≥120 ms), various RsR′ patterns were defined as fQRS depending on the presence of >2 R′ waves or >2 notches in the R or S waves in two contiguous leads (Figure 1).

The strain pattern was defined as a descending ST segment depression of ≥1 mm with an inverted asymmetrical T wave opposite to the QRS axis in at least two contiguous leads (Lin et al., 2013).

### 2.4 Statistical analysis

The normal distribution of data was investigated with the Kolmogorov–Smirnov test. The characteristics of groups were compared with an independent *t* test or Mann–Whitney test, as appropriate. The sensitivity of the different hypertrophy indices was compared with the McNemar test. The correlation between continuous variables was calculated with Spearman's correlation analysis. Multiple regression analysis was performed to identify predictors of myocardial fibrosis. The interobserver agreement was examined with the intraclass correlation coefficient (ICC score). An ICC of less than 0.4 was considered poor; an ICC of 0.4–0.75 was considered fair to good; an ICC of greater than 0.75 was considered excellent. Differences were considered statistically significant when *p* < .05. All analyses were performed by using MedCalc software (version 17.9.5).

## 3 RESULTS

### 3.1 Patient characteristics

The HCM patients had the following symptoms: syncope (18%), chest pain (40%), dyspnea (32%), and palpitation (30%). One patient had a history of sustained ventricular tachycardia, and one patient had a history of aborted sudden cardiac death. None of the examined HCM patients had an implanted cardioverter defibrillator device at the time of the CMR examination. HCM was the referral diagnosis in 90% of the cases based on echocardiographic and ECG findings. There was no difference between HCM patients and the control group in age (mean age of HCM patients: 49, *SD*: 17; mean age of controls: 44, *SD*: 8; *p* = .07), in the gender ratio (percent males in the HCM group: 60%; in the control group: 54%; *p* = .52), and in the BSA (mean BSA of HCM patients: 1.96, *SD*: 0.26; mean BSA of controls: 1.90, *SD*: 0.22; *p* = .19). The CMR and ECG characteristics of the study population are summarized in Table 1. In the control group, no pathological alterations were found with CMR. In HCM patients, asymmetric hypertrophy with a septal or an anterior distribution was found in 108 (74%) patients. There were 24 (16%) patients with apical HCM, 11 (8%) patients with concentric HCM, and three (2%) patients with midventricular HCM. Myocardial fibrosis was present in 90% of patients. More extensive fibrosis was found in patients with a higher LVM (*p* < .0001; *r* = .486) and maximal end‐diastolic wall thickness (*p* < .0001; *r* = .576). There was no burned‐out HCM with a severely reduced left ventricular ejection fraction in this population.

**TABLE 1:** CMR and ECG characteristics of the study population

Number of patients	HCM		Control group		*p*
	146		35		
CMR parameters	Mean	*SD*	Mean	*SD*	
LVEF (%)	64	7	62	5	.19
LVESVi (ml/m^2^)	31	8	34	13	.23
LVEDVi (ml/m^2^)	84	15	84	11	.93
LVSVi (ml/m^2^)	53	11	52	6	.53
LVM (g)	171	67	87	25	<.0001
LVMi (g/m^2^)	87	32	46	10	<.0001
Maximal wall thickness (mm)	20	5	9	2	<.0001
Myocardial fibrosis (g)	17	22	—		
Myocardial fibrosis (%)	9	10	—		

ECG parameters	Number	%	Number	%	
Pathological Q waves	36	25	0	0	.0011
fQRS	71	49	6	17	.0007
ST depression	94	64	0	0	<.0001
ST elevation	35	24	0	0	.0013
T‐wave inversion	116	80	1	3	<.0001
Strain pattern	74	51	0	0	<.0001
Sokolow–Lyon index positivity	46		1		.0005
	Sensitivity 32%, specificity 97%, AUC 0.644				
Cornell index positivity	51		0		<.0001
	Sensitivity 35%, specificity 100%, AUC 0.675				
Romhilt–Estes score ≥5	90		0		<.0001
	Sensitivity 62%, specificity 100%, AUC 0.808				
Romhilt–Estes score ≥4	109		1		<.0001
	Sensitivity 75%, specificity 97%, AUC 0.859				

Abbreviations: CMR, cardiac magnetic resonance; ECG, electrocardiograph; EDVi, end‐diastolic volume index; EF, ejection fraction; ESVi, end‐systolic volume index; fQRS, fragmented QRS; HCM, hypertrophic cardiomyopathy; LV, left ventricular; LVMi, left ventricular mass index; SD, standard deviation; SVi, stroke volume index.

Hypertrophic cardiomyopathy patients had a significantly higher LVM and maximal wall thickness than individuals in the control group. All of the investigated ECG alterations occurred significantly more frequently in patients with HCM (Table 1).

### 3.2 Diagnostic accuracy of the different ECG hypertrophy criteria

The sensitivity and specificity of the ECG hypertrophy criteria are summarized in Table 1. All three ECG hypertrophy criteria had a high specificity. A total of 126 (86%) HCM patients met at least one of the three investigated ECG hypertrophy criteria. The Sokolow–Lyon index was positive in 46 (32%) patients, and the Cornell index was positive in 51 (35%) patients. The Romhilt–Estes score suggested definitive LVH (a Romhilt–Estes score ≥5) in 90 (62%) patients and probable LVH (a Romhilt–Estes score = 4) in 19 (13%) additional patients. Comparing the sensitivity of the three investigated the ECG voltage criteria, the Romhilt–Estes score was the most sensitive (Romhilt–Estes score vs. Sokolow–Lyon index: difference 43.2%, 95% confidence interval 33.7% to 52.6%, *p* < .0001; Romhilt–Estes score vs. Cornell index: difference 39.7%, 95% confidence interval 29.6% to 49.9%, *p* < .0001). There was no statistically significant difference in the sensitivity of the Sokolow–Lyon index and Cornell index (difference: 3.4%, 95% confidence interval −7.1% to 13.9%, *p* = .61).

**FIGURE 2:**
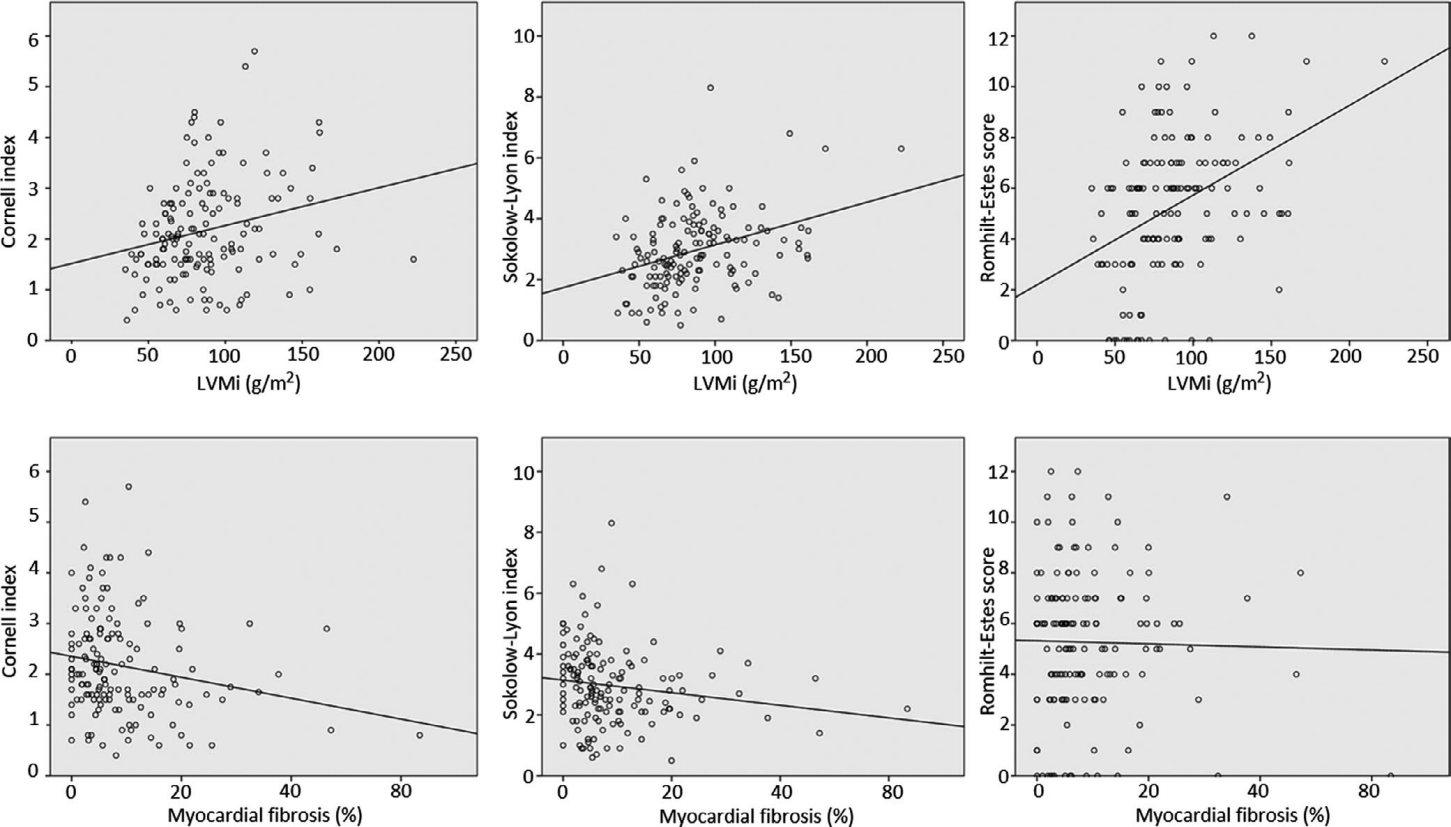
Correlations between the ECG hypertrophy criteria and LVMi, the ECG hypertrophy criteria, and myocardial fibrosis (Spearman's correlation). ECG, electrocardiograph; LVMi, left ventricular mass index

The left ventricular mass index (LVMi) was positively correlated with the Cornell index (*p* = .0018; *r* = .257), the Sokolow–Lyon index (*p* < .0001; *r* = .337), and the Romhilt–Estes score (*p* < .0001; *r* = .410), which had the strongest correlation (Figure 2).

### 3.3 Effect of fibrosis on the voltage criteria

The amount of fibrosis was negatively correlated with the Cornell index (*p* = .015; *r* = −.201) and the Sokolow–Lyon index (*p* = .0052; *r* = −.230). The Romhilt–Estes score was independent of the amount of fibrosis (*p* = .757; *r* = .026) (Figure 2).

### 3.4 ECG predictors of the LVM and myocardial fibrosis

When we compared the LVM and the amount of fibrosis in HCM patients with and without an ECG abnormality, we found that patients with ST depression, T‐wave inversion, strain pattern, Sokolow–Lyon index positivity, or Romhilt–Estes score positivity had a significantly higher LVM. The amount of myocardial fibrosis was significantly higher in patients with fQRS or strain pattern and was lower in patients with Sokolow–Lyon index positivity. No significant differences in LVM or the amount of myocardial fibrosis were found if a pathological Q wave, ST elevation, or Cornell index positivity was present (Table 2).

**TABLE 2:** Amount of myocardial fibrosis and the LVM in patients with and without an ECG abnormality

	ECG abnormality present			ECG abnormality absent			Fibrosis *p*	LVM *p*
	*n*	Fibrosis (%) median (interquartile range)	LVM (g) median (interquartile range)	*n*	Fibrosis (%) median (interquartile range)	LVM (g) median (interquartile range)		
Pathological Q waves	36	7.2 (4.4–12.4)	160 (123–225)	110	5.7 (2.6–11.7)	158 (131–200)	.13	.92
fQRS	71	8.0 (4.4–14.5)	170 (131–212)	75	5.0 (2.6–8.6)	152 (120–187)	**.0015**	.11
ST depression	94	6.6 (3.2–11.2)	175 (131–221)	52	5.2 (2.7–13.8)	145 (115–177)	.50	**.0016**
ST elevation	35	5.7 (2.8–12.4)	162 (127–191)	111	6.2 (3.4–11.1)	157 (129–207)	.83	.66
T‐wave inversion	116	6.2 (3.0–12.0)	162 (131–211)	30	6.0 (3.1–10.2)	135 (117–182)	.87	**.0437**
Strain pattern	74	6.9 (4.1–14.0)	175 (136–223)	72	5.2 (2.4–9.7)	146 (113–187)	**.0174**	**.0032**
Sokolow–Lyon index positivity	46	4.5 (1.9–7.1)	175 (146–213)	100	6.9 (3.8–14.0)	149 (118–194)	**.0023**	**.0273**
Cornell index positivity	51	5.4 (2.6–8.8)	174 (139–216)	95	6.2 (3.2–12.7)	157 (124–194)	.24	.16
Romhilt–Estes score ≥ 4	109	6.3 (3.4–12.7)	174 (138–214)	37	5.0 (2.4–10.3)	129 (91–153)	.20	**<.0001**
Romhilt–Estes score ≥ 5	90	6.1 (3.2–12.2)	175 (142–217)	56	5.9 (3.0–11.0)	140 (107–179)	.82	**.0002**

Note

Patients with fQRS or strain pattern had more myocardial fibrosis, and patients with Sokolow–Lyon index positivity had less myocardial fibrosis. Patients with ST depression, T‐wave inversion, strain pattern, Sokolow–Lyon index positivity, or Romhilt–Estes score positivity had a significantly higher LVM.

Abbreviations: ECG, electrocardiograph; fQRS, fragmented QRS; LVM, left ventricular ejection fraction, volumes, and myocardial mass.

Bold values indicate the significant differences and predictors.

Based on the multivariate analysis, we found that male gender, the strain pattern, the Sokolow–Lyon index, and the Romhilt–Estes score were independent positive predictors of LVM (*p* < .0001). fQRS and the strain pattern predicted more fibrosis, while the Cornell index was a negative predictor of myocardial fibrosis (*p* < .0001) (Table 3).

**TABLE 3:** ECG predictors of the LVM and myocardial fibrosis

	ECG predictors of the LVM (g)				ECG predictors of myocardial fibrosis (%)			
	Univariate analysis		Multivariate analysis		Univariate analysis		Multivariate analysis	
	Coefficient	*p*	Coefficient	*p*	Coefficient	*p*	Coefficient	*p*
(Constant)			74.46				9.26	
Age	−0.82	**.016**			−0.07	.16		
Male gender	61.77	**<.0001**	53.79	<.0001	−0.81	.63		
Pathological Q waves	7.57	.56			0.88	.64		
fQRS	21.44	.054			5.38	**.0008**	4.58	.0032
ST depression	36.40	**.0015**			−0.58	.73		
ST elevation	−11.03	.40			−0.95	.62		
T‐wave inversion	24.72	.07			1.04	.61		
Strain pattern	32.93	**.0028**	19.48	.045	3.93	**.015**	4.05	.0095
Sokolow–Lyon index	14.83	**.0005**	9.28	.014	−1.20	.058		
Cornell index	13.11	**.0174**			−1.94	**.016**	−2.05	.008
Romhilt–Estes score	8.32	**<.0001**	5.05	.005	−0.07	.80		

Note

The male gender, the presence of strain pattern, a 1‐mV increase in the Sokolow–Lyon index, or a one‐point increase in the Romhilt–Estes score independently predicted 54, 19, 9 and 5 g increases in the LVM, respectively. The presence of fQRS or strain patterns independently predicted an additional 4.58% and 4.05% of fibrotic area in the myocardium, respectively. A 1‐mV increase in the Cornell index predicted a 2.05% decrease in myocardial fibrosis.

Abbreviations: ECG, electrocardiograph; fQRS, fragmented QRS; LVM, left ventricular ejection fraction, volumes, and myocardial mass.

Bold values indicate the significant differences and predictors.

### 3.5 Interobserver agreement

In the investigation of the interobserver variability in LVM measurements and myocardial fibrosis quantifications, the interobserver agreement was excellent for all parameters (ICC of LVM 0.976, myocardial fibrosis (g) 0.966, and myocardial fibrosis (%) 0.951).

## 4 DISCUSSION

The diagnostic value of the different ECG voltage criteria for HCM is mainly investigated using echocardiography (Chen et al., 2014; Erice et al., 2009; Grossman, Prokupetz, Koren‐Morag, Grossman, & Shamiss, 2012). Although CMR is the gold standard noninvasive method for the detection and quantification of myocardial fibrosis, limited CMR data are available. Hypertrophy and myocardial fibrosis both have an effect on ECGs, and an increased myocardial mass result in a higher ECG amplitude, but replacement of the myocardium by fibrotic tissue decreases the ECG voltage. Based on the results from the Multi‐Ethnic Study of Atherosclerosis, diffuse myocardial fibrosis was associated with a lower QRS voltage in a large population free of clinical cardiovascular diseases (Inoue et al., 2017). It is also known that end‐stage HCM with extensive fibrosis is associated with low voltage on ECGs (Konno et al., 2016). These studies did not investigate the effect of fibrosis on the Romhilt–Estes score, which is a more complex criterion than the other ECG hypertrophy criteria. In our study, the Romhilt–Estes score was the most sensitive ECG hypertrophy criterion, and this criterion showed the strongest correlation with the LVM. The Romhilt–Estes score was independent of the extent of fibrosis. The explanation that we offer for this finding is that the Romhilt–Estes score considers not only voltage criteria but also ST‐T abnormalities, P‐wave features, left axis deviations, QRS durations and delayed intrinsicoid deflections; thus, the complexity of this score may result in a higher diagnostic accuracy in HCM.

Although pathological Q waves are traditionally considered a marker of myocardial scarring, we found no difference in the amount of fibrosis between patients with and without pathological Q waves. In contrast to that result, patients with fQRS or strain pattern had significantly higher amounts of fibrosis, and fQRS and/or strain pattern predicted myocardial fibrosis. In previous studies, it was also found that the presence of fQRS might be correlated with more fibrosis (Konno et al., 2015; Park et al., 2018). Other studies that investigated the prognostic significance of fQRS reported that the presence of fQRS was associated with a significant increase in arrhythmic events in HCM patients (Femenia et al., 2013; Ozyilmaz et al., 2017). Strain pattern is a known ECG sign of HCM and is associated with a higher cardiovascular risk and abnormal left ventricular function (Goldberger, 1979; Nomura et al., 2018; Ogah et al., 2008). It is also known that ECG strain is a marker of myocardial fibrosis in aortic stenosis and in hypertension (Rodrigues et al., 2017; Shah et al., 2014). To our knowledge, how strain pattern predicts myocardial fibrosis was not previously investigated in patients with HCM. These results suggest that the presence of fQRS and/or strain pattern may indicate a greater amount of myocardial fibrosis and a higher risk in patients with HCM.

Fragmented QRS is a relatively common ECG alteration in the normal population. In our control group, 17% of healthy individuals had fQRS. A similar prevalence was found in a Finnish study, and fQRS was present in 19.7% of a middle‐aged general population consisting of 10,904 subjects. In this study, the prognostic significance of fQRS was investigated, and the researchers found that fQRS was not associated with increased mortality in subjects without a known cardiac disease (Terho et al., 2014).

### 4.1 Study limitations

A limitation of our study is that it was a single‐center study with a relatively small control group. As non‐contrast CMR examinations were performed in the control group because of ethical considerations, the presence of myocardial fibrosis was unknown in this healthy study population. Myocardial T1 and T2 mapping and myocardial extracellular volume evaluations were not available. Another limitation is that there was no follow‐up of the patients in this study, and no genetic testing was performed in HCM patients.

## 5 CONCLUSION

Our results suggest that in patients with HCM, the Romhilt–Estes score detects LVH with the highest sensitivity, as myocardial fibrosis has no effect on this criterion. In contrast to pathological Q waves, fQRS and strain pattern are reliable predictors of myocardial fibrosis.

## CONFLICT OF INTEREST

The authors have stated explicitly that there are no conflicts of interest in connection with this article.

## AUTHOR CONTRIBUTIONS

Zsofia Dohy, Andras Vereckei, Viktor Horvath, Csilla Czimbalmos, Liliana Szabo, Attila Toth, Ferenc I. Suhai, Ibolya Csecs, David Becker, Bela Merkely and Hajnalka Vago: takes responsibility for all aspects of the reliability and freedom from bias of the data presented and their discussed interpretation.

## ETHICS

Ethical approval was obtained from the Hungarian National Institute of Pharmacy and Nutrition (OGYEI/29174‐4/2019), and this study was performed in accordance with the ethical standards in the 1964 Declaration of Helsinki and its later amendments.
